# The Ontario Veterinary Association

**Published:** 1901-01

**Authors:** C. H. Sweetapple

**Affiliations:** Secretary-Treasurer


### THE ONTARIO VETERINARY ASSOCIATION.

THE annual meeting of this Association was held in the Ontario Veterinary College, Toronto, Canada, on Friday, December 21, 1900.

The President, Mr. W. J. Wilson, V.S., of London, Ont., opened the meeting with a short address, the substance of which was as follows: "The assembling of our members to-day in annual convention is worthy of more than a passing notice, as it marks the closing of the nineteenth century, which has been one of wonderful advancement in veterinary science. At the beginning of the century veterinary surgeons were almost unheard of and our literature was very limited, whereas to-day we hold an honorable position among the professions of the world and our literature is very extensive. I am proud to say that the Ontario Veterinary College has kept well to the front. I believe it to be equal in its facilities for instruction to any veterinary college on the continent of America. I am also proud to say that its graduates as a body have well sustained the reputation of their alma mater. This Association was organized in the year 1874. It has continued to meet periodically ever since its organization, and its original objects have been constantly kept in view, namely, the mutual improvement of its members in those branches of knowledge specially pertaining to their profession and the advancement of the position and interests of the veterinary profession in the province of Ontario."

The minutes of the previous meeting were read and confirmed.

The Treasurer's and Auditor's reports were read and confirmed.

The Secretary reported the failure of the endeavorers to get the act passed for better legislative measures for the protection of the profession.

The Secretary also reported three convictions secured by the prosecutor for violations of the present veterinary act.

A committee was appointed to revise the By-laws of the Association, and the meeting adjourned for luncheon.

After luncheon an animated discussion at once commenced, in which many members took part, relating to some alleged violations of professional ethics, and the Committee on Revision of the By-laws reported "that our present By-laws be amended by introducing a by-law to prevent members of this Association engaging in the preparation of any proprietary medicine and placing the same upon the market, and also that any member engaged in preparing secret formulæ and selling, handling, and disposing of the same shall by the provisions of this by-law be disqualified from holding any office in the gift of this Association."

This report of the committee was adopted.

Mr. Shillingham gave an account of a rather peculiar case, in a mare, of very painful, sensitive swellings appearing sometimes after parturition, these swellings disappearing and recurring again in different parts of the body at intervals, and the mare otherwise in apparent good health.

Mr. Tennent still finds most excellent results from Schmidt's treatment of so-called parturient apoplexy of the cow, as he reported last year. He adds a little formalin to the iodide of potassium solution.

Mr. McKay and others also reported very strongly in favor of Schmidt's treatment.

Professor Reed, of Guelph Agricultural College, does not believe in Schmidt's treatment. He considers its favorable results are due to abstaining from drenching the cow while in a comatose state. He always gives his medicine through a tube passed down the œsophagus. He believes in chloral hydrate and potassium bromide and nerve stimulants.

The sum of $25 was appropriated for a gold medal to be competed for by the graduating class of the Ontario Veterinary College at the coming spring examinations.

The following officers were elected for the ensuing year: H. S. Wende, President; J. H. Tennent, First Vice-President; W. Steele, Second Vice-President; C. H. Sweetapple, Secretary and Treasurer. Directors: F. G. Hutton, J. H. George, J. Wagner, W. Shillinglaw, F. J. Gallanough, W. Lawson, D. McMurtry, and S. E. Boulter. Auditors: C. Elliott and J. H. Reed. Delegates to Industrial Fair, Toronto: Prof. A. Smith and Dr. Duncan. Delegates to the Western Fair, London: W. J. Wilson and J. H. Tennent.

C. H. SWEETAPPLE, V.S.,
Secretary-Treasurer.

